# Crystal structure of *N*-(propan-2-yl­carbamo­thio­yl)benzamide

**DOI:** 10.1107/S2056989014027133

**Published:** 2015-01-01

**Authors:** Jerry P. Jasinski, Mehmet Akkurt, Shaaban K. Mohamed, Mohamed A. Gad, Mustafa R. Albayati

**Affiliations:** a Department of Chemistry, Keene State College, 229 Main Street, Keene, NH 03435-2001, USA; b Department of Physics, Faculty of Sciences, Erciyes University, 38039 Kayseri, Turkey; c Chemistry and Environmental Division, Manchester Metropolitan University, Manchester M1 5GD, England; d Chemistry Department, Faculty of Science, Minia University, 61519 El-Minia, Egypt; e Chemistry Department, Faculty of Science, Sohag University, 82524 Sohag, Egypt; f Kirkuk University, College of Science, Department of Chemistry, Kirkuk, Iraq

**Keywords:** crystal structure, thio­urea, conformation, hydrogen bonding

## Abstract

In the crystal structure of the title compound, C_11_H_14_N_2_OS, the six atoms of the central C_2_N_2_OS residue are coplanar (r.m.s. deviation = 0.002 Å), which facilitates the formation of an intra­molecular N—H⋯O hydrogen bond, which closes an *S*(6) loop. The terminal phenyl ring is inclined with respect to the central plane [dihedral angle = 42.10 (6)°]. The most prominent feature of the crystal packing is the formation of {⋯HNCS}_2_ synthons resulting in centrosymmetric dimers.

## Related literature   

For use of thio­ureas as building blocks in the synthesis of various organic compounds, see: Burgeson *et al.* (2012 ▸); Vega-Pérez *et al.* (2012 ▸); Yao *et al.* (2012 ▸); Shantharam *et al.* (2013 ▸); Yang *et al.* (2013 ▸). For use of thio­urea-containing compounds in medicinal applications, see: Rodriguez-Fernandez *et al.* (2005 ▸); Rauf *et al.* (2012 ▸).
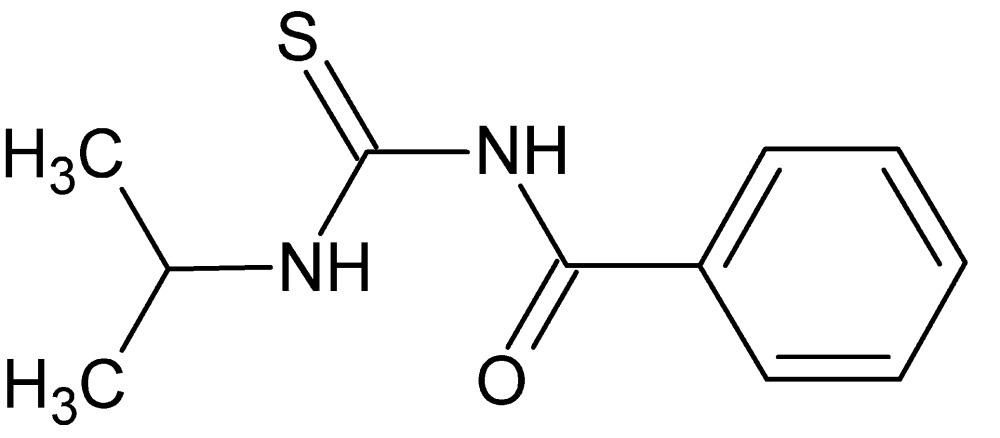


## Experimental   

### Crystal data  

C_11_H_14_N_2_OS
*M* *_r_* = 222.30
Monoclinic, 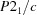

*a* = 11.2147 (4) Å
*b* = 5.3988 (2) Å
*c* = 19.6834 (7) Å
β = 102.031 (4)°
*V* = 1165.57 (7) Å^3^
*Z* = 4
Cu *K*α radiation
μ = 2.27 mm^−1^
*T* = 293 K
0.28 × 0.22 × 0.18 mm

### Data collection  

Agilent Xcalibur, Eos, Gemini diffractometer
Absorption correction: multi-scan (*CrysAlis PRO*; Agilent, 2014 ▸) *T*_min_ = 0.828, *T*_max_ = 1.000
3844 measured reflections
2189 independent reflections
1944 reflections with *I* > 2σ(*I*)
*R*_int_ = 0.025

### Refinement  

*R*[*F* ^2^ > 2σ(*F* ^2^)] = 0.049
*wR*(*F* ^2^) = 0.146
*S* = 1.08
2189 reflections
146 parameters
2 restraints
H atoms treated by a mixture of independent and constrained refinement
Δρ_max_ = 0.37 e Å^−3^
Δρ_min_ = −0.34 e Å^−3^

### 

Data collection: *CrysAlis PRO* (Agilent, 2014 ▸); cell refinement: *CrysAlis PRO*; data reduction: *CrysAlis PRO*; program(s) used to solve structure: *SHELXS2014* (Gruene *et al.*, 2014 ▸); program(s) used to refine structure: *SHELXL2014* (Gruene *et al.*, 2014 ▸); molecular graphics: *ORTEP-3 for Windows* (Farrugia, 2012 ▸); software used to prepare material for publication: *PLATON* (Spek, 2009 ▸).

## Supplementary Material

Crystal structure: contains datablock(s) global, I. DOI: 10.1107/S2056989014027133/tk5351sup1.cif

Structure factors: contains datablock(s) I. DOI: 10.1107/S2056989014027133/tk5351Isup2.hkl

Supporting information file. DOI: 10.1107/S2056989014027133/tk5351Isup3.cml

**Fig. 1.**
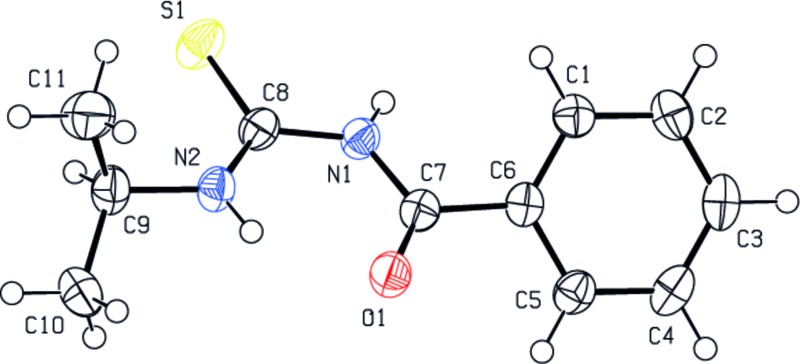
Perspective view of the title molecule with atom labeling scheme and 50% probability ellipsoids.

**Fig. 2.**
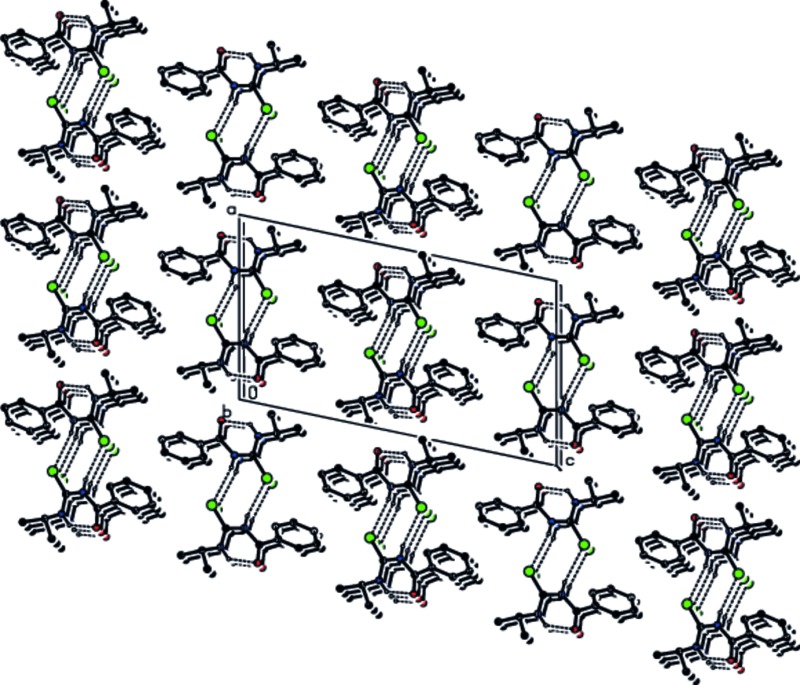
Packing viewed down the b axis showing stacks of pairs of molecules connected by N—H···S interactions.

. DOI: 10.1107/S2056989014027133/tk5351fig1.tif
Perspective view of the title mol­ecule with atom labeling scheme and 50% probability ellipsoids.

b . DOI: 10.1107/S2056989014027133/tk5351fig2.tif
Packing viewed down the *b* axis showing stacks of pairs of mol­ecules connected by N—H⋯S inter­actions.

CCDC reference: 1038725

Additional supporting information:  crystallographic information; 3D view; checkCIF report

## Figures and Tables

**Table 1:** Hydrogen-bond geometry (, )

*D*H*A*	*D*H	H*A*	*D* *A*	*D*H*A*
N1H1*N*S1^i^	0.81(2)	2.66(2)	3.4439(19)	165(2)
N2H2*N*O1	0.87(2)	2.00(3)	2.662(2)	132(2)

Symmetry code: (i) 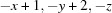.

## supplementary crystallographic information

### S1. Structural commentary

Compounds containing thio­urea linkage are very useful building blocks for the synthesis of a wide range of multiheterocyclic and macromolecular compounds. Thio­ureas have proved to be useful substances in drug research in recent years (Burgeson *et al.*, 2012; Vega-Pérez *et al.*, 2012; Yao *et al*., 2012; Shantharam *et al.*, 2013; Yang *et al.*, 2013). Symmetrical and unsymmetrical thio­ureas have shown anti-fungal activity against the plant pathogens like *Penicillium expansum* and *Fusarium oxysporu*m (Rodriguez-Fernandez *et al.*, 2005). Also, 1,3-di­alkyl or di­aryl thio­ureas exhibited significant anti-fungal activity against *Pyricularia oryzae* and *Drechslera oryzae* (Rauf *et al.*, 2012). In light of this, and following to our on-going study in synthesis of bio-active molecules, we report here the synthesis and crystal structure of the title compound.

In the structure of the title compound, Fig. 1, intra­molecular N—H···O and inter­molecular N—H···S inter­actions are noted (Table 1).

### S2. Synthesis and crystallization

Freshly prepared benzoyl chloride 5 ml (0.043 mol) was added drop wise to a solution of 3.2 g (0.042 mol) of ammonium thio­cyanate in 20 ml dry acetone with stirring. The reaction mixture was refluxed for 3 h. The obtained solid precipitate ammonium chloride was filtered off. The formed benzoyl iso­thio­cyanate in the filtrate was added to a solution of 3.1 ml (0.0425 mol) of 2-amino-iso­propane in 20 ml dry acetone. The reaction mixture was heated under reflux for 5 h, then poured into a beaker containing some ice cubes. The resulting precipitate was collected by filtration, washed several times with cold ethanol/water and purified by recrystallization from ethanol/di­chloro­methane mixture (1:1). Yield (63%); colourless solid, m.p 418 K.

### S3. Refinement

Carbon-bound H-atoms were placed in calculated positions (C—H = 0.93 to 0.98 Å) and were included in the refinement in the riding model approximation, with *U_iso_*(H) = 1.2–1.5*U_eq_*(C). The hydrogen atoms attached to N1 and N2 were found from difference Fourier maps and were refined with the distance contratin N—H = 0.86±0.02 Å with unrestrained *U*_iso_.

### Crystal data

**Table:**

C_11_H_14_N_2_OS	*F*(000) = 472
*M**_r_* = 222.30	*D*_x_ = 1.267 Mg m^−^^3^
Monoclinic, *P*2_1_/*c*	Cu *K*α radiation, λ = 1.54184 Å
Hall symbol: -P 2ybc	Cell parameters from 1804 reflections
*a* = 11.2147 (4) Å	θ = 4.0–71.4°
*b* = 5.3988 (2) Å	µ = 2.27 mm^−^^1^
*c* = 19.6834 (7) Å	*T* = 293 K
β = 102.031 (4)°	Prism, colourless
*V* = 1165.57 (7) Å^3^	0.28 × 0.22 × 0.18 mm
*Z* = 4	

### Data collection

**Table:**

Agilent Xcalibur, Eos, Gemini diffractometer	2189 independent reflections
Radiation source: Enhance (Cu) X-ray Source	1944 reflections with *I* > 2σ(*I*)
Graphite monochromator	*R*_int_ = 0.025
Detector resolution: 16.0416 pixels mm^-1^	θ_max_ = 71.3°, θ_min_ = 4.0°
ω scans	*h* = −12→13
Absorption correction: multi-scan (*CrysAlis PRO*; Agilent, 2014)	*k* = −6→5
*T*_min_ = 0.828, *T*_max_ = 1.000	*l* = −18→24
3844 measured reflections	

### Refinement

**Table:**

Refinement on *F*^2^	2 restraints
Least-squares matrix: full	Hydrogen site location: mixed
*R*[*F*^2^ > 2σ(*F*^2^)] = 0.049	H atoms treated by a mixture of independent and constrained refinement
*wR*(*F*^2^) = 0.146	*w* = 1/[σ^2^(*F*_o_^2^) + (0.0871*P*)^2^ + 0.3862*P*] where *P* = (*F*_o_^2^ + 2*F*_c_^2^)/3
*S* = 1.08	(Δ/σ)_max_ < 0.001
2189 reflections	Δρ_max_ = 0.37 e Å^−^^3^
146 parameters	Δρ_min_ = −0.34 e Å^−^^3^

### Special details

**Table:**

Geometry. Bond distances, angles *etc*. have been calculated using the rounded fractional coordinates. All su's are estimated from the variances of the (full) variance-covariance matrix. The cell e.s.d.'s are taken into account in the estimation of distances, angles and torsion angles
Refinement. Refinement on *F*^2^ for ALL reflections except those flagged by the user for potential systematic errors. Weighted *R*-factors *wR* and all goodnesses of fit *S* are based on *F*^2^, conventional *R*-factors *R* are based on *F*, with *F* set to zero for negative *F*^2^. The observed criterion of *F*^2^ > σ(*F*^2^) is used only for calculating -*R*-factor-obs *etc*. and is not relevant to the choice of reflections for refinement. *R*-factors based on *F*^2^ are statistically about twice as large as those based on *F*, and *R*-factors based on ALL data will be even larger.

### Fractional atomic coordinates and isotropic or equivalent isotropic displacement parameters (Å^2^)

**Table:**

	*x*	*y*	*z*	*U*_iso_*/*U*_eq_	
S1	0.60730 (5)	0.80543 (14)	0.08368 (3)	0.0464 (2)	
O1	0.86531 (13)	1.0046 (3)	−0.05679 (8)	0.0392 (5)	
N1	0.69070 (16)	1.0345 (3)	−0.01498 (9)	0.0321 (5)	
N2	0.82704 (16)	0.7717 (3)	0.05595 (9)	0.0337 (5)	
C1	0.63795 (19)	1.4665 (4)	−0.10695 (11)	0.0334 (6)	
C2	0.5930 (2)	1.6272 (4)	−0.16095 (12)	0.0392 (6)	
C3	0.6171 (2)	1.5865 (4)	−0.22650 (11)	0.0414 (7)	
C4	0.6865 (2)	1.3853 (5)	−0.23779 (11)	0.0402 (7)	
C5	0.73328 (19)	1.2248 (4)	−0.18386 (11)	0.0342 (6)	
C6	0.70827 (17)	1.2641 (4)	−0.11803 (10)	0.0293 (5)	
C7	0.76319 (18)	1.0900 (4)	−0.06135 (10)	0.0310 (6)	
C8	0.71668 (19)	0.8697 (4)	0.04084 (10)	0.0322 (6)	
C9	0.8684 (2)	0.5923 (4)	0.11200 (11)	0.0389 (7)	
C10	0.9666 (2)	0.4305 (4)	0.09293 (13)	0.0452 (7)	
C11	0.9154 (3)	0.7308 (6)	0.17988 (13)	0.0592 (9)	
H1	0.62120	1.49370	−0.06320	0.0400*	
H1N	0.6216 (16)	1.085 (4)	−0.0236 (11)	0.022 (5)*	
H2	0.54640	1.76300	−0.15340	0.0470*	
H2N	0.880 (2)	0.814 (5)	0.0317 (14)	0.048 (8)*	
H3	0.58660	1.69470	−0.26270	0.0500*	
H4	0.70190	1.35740	−0.28180	0.0480*	
H5	0.78120	1.09110	−0.19140	0.0410*	
H9	0.79950	0.48790	0.11730	0.0470*	
H10A	0.93400	0.34290	0.05070	0.0680*	
H10B	0.99470	0.31390	0.12960	0.0680*	
H10C	1.03360	0.53220	0.08640	0.0680*	
H11A	0.98180	0.83660	0.17480	0.0890*	
H11B	0.94320	0.61370	0.21650	0.0890*	
H11C	0.85090	0.82920	0.19110	0.0890*	

### Atomic displacement parameters (Å^2^)

**Table:**

	*U*^11^	*U*^22^	*U*^33^	*U*^12^	*U*^13^	*U*^23^
S1	0.0297 (3)	0.0743 (5)	0.0364 (3)	0.0061 (2)	0.0096 (2)	0.0129 (3)
O1	0.0309 (8)	0.0465 (9)	0.0415 (8)	0.0072 (6)	0.0106 (6)	0.0113 (7)
N1	0.0278 (8)	0.0390 (9)	0.0293 (8)	0.0046 (7)	0.0056 (6)	0.0054 (7)
N2	0.0306 (9)	0.0383 (10)	0.0314 (9)	0.0010 (7)	0.0046 (7)	0.0076 (7)
C1	0.0350 (10)	0.0309 (10)	0.0331 (10)	−0.0033 (8)	0.0041 (8)	−0.0023 (8)
C2	0.0400 (11)	0.0299 (10)	0.0448 (12)	−0.0012 (9)	0.0019 (9)	0.0025 (9)
C3	0.0418 (12)	0.0405 (12)	0.0377 (11)	−0.0059 (9)	−0.0014 (9)	0.0124 (9)
C4	0.0369 (11)	0.0544 (14)	0.0291 (10)	−0.0081 (10)	0.0064 (8)	0.0062 (9)
C5	0.0284 (10)	0.0408 (11)	0.0341 (10)	−0.0027 (8)	0.0080 (8)	0.0032 (8)
C6	0.0260 (9)	0.0304 (9)	0.0300 (9)	−0.0055 (7)	0.0022 (7)	0.0023 (8)
C7	0.0321 (10)	0.0314 (10)	0.0286 (9)	−0.0038 (8)	0.0043 (7)	−0.0007 (8)
C8	0.0336 (10)	0.0363 (10)	0.0255 (9)	−0.0020 (8)	0.0033 (7)	−0.0004 (8)
C9	0.0367 (11)	0.0412 (12)	0.0379 (11)	0.0022 (9)	0.0059 (9)	0.0114 (9)
C10	0.0487 (13)	0.0347 (11)	0.0506 (13)	0.0075 (10)	0.0065 (10)	0.0043 (10)
C11	0.0607 (16)	0.079 (2)	0.0334 (12)	0.0280 (14)	−0.0003 (11)	0.0010 (12)

### Geometric parameters (Å, º)

**Table:**

S1—C8	1.664 (2)	C9—C11	1.526 (3)
O1—C7	1.220 (3)	C9—C10	1.513 (3)
N1—C7	1.376 (3)	C1—H1	0.9300
N1—C8	1.397 (3)	C2—H2	0.9300
N2—C8	1.322 (3)	C3—H3	0.9300
N2—C9	1.469 (3)	C4—H4	0.9300
C1—C2	1.383 (3)	C5—H5	0.9300
C1—C6	1.391 (3)	C9—H9	0.9800
N1—H1N	0.806 (19)	C10—H10A	0.9600
N2—H2N	0.87 (2)	C10—H10B	0.9600
C2—C3	1.390 (3)	C10—H10C	0.9600
C3—C4	1.381 (3)	C11—H11A	0.9600
C4—C5	1.386 (3)	C11—H11B	0.9600
C5—C6	1.398 (3)	C11—H11C	0.9600
C6—C7	1.490 (3)		
			
C7—N1—C8	127.26 (18)	C6—C1—H1	120.00
C8—N2—C9	124.57 (18)	C1—C2—H2	120.00
C2—C1—C6	120.0 (2)	C3—C2—H2	120.00
C7—N1—H1N	117.5 (15)	C2—C3—H3	120.00
C8—N1—H1N	114.5 (15)	C4—C3—H3	120.00
C1—C2—C3	120.3 (2)	C3—C4—H4	120.00
C8—N2—H2N	119.2 (17)	C5—C4—H4	120.00
C9—N2—H2N	116.2 (17)	C4—C5—H5	120.00
C2—C3—C4	119.9 (2)	C6—C5—H5	120.00
C3—C4—C5	120.3 (2)	N2—C9—H9	109.00
C4—C5—C6	119.9 (2)	C10—C9—H9	109.00
C1—C6—C7	122.51 (18)	C11—C9—H9	109.00
C5—C6—C7	117.82 (18)	C9—C10—H10A	109.00
C1—C6—C5	119.63 (19)	C9—C10—H10B	110.00
O1—C7—N1	122.96 (19)	C9—C10—H10C	109.00
O1—C7—C6	121.90 (18)	H10A—C10—H10B	110.00
N1—C7—C6	115.14 (17)	H10A—C10—H10C	109.00
S1—C8—N1	118.47 (16)	H10B—C10—H10C	109.00
S1—C8—N2	123.87 (16)	C9—C11—H11A	110.00
N1—C8—N2	117.65 (19)	C9—C11—H11B	109.00
N2—C9—C11	109.39 (19)	C9—C11—H11C	109.00
C10—C9—C11	111.3 (2)	H11A—C11—H11B	109.00
N2—C9—C10	109.06 (18)	H11A—C11—H11C	109.00
C2—C1—H1	120.00	H11B—C11—H11C	110.00
			
C7—N1—C8—N2	7.1 (3)	C2—C1—C6—C7	177.6 (2)
C7—N1—C8—S1	−172.18 (17)	C1—C2—C3—C4	−0.2 (3)
C8—N1—C7—O1	−3.3 (3)	C2—C3—C4—C5	−0.6 (3)
C8—N1—C7—C6	176.95 (19)	C3—C4—C5—C6	1.2 (3)
C9—N2—C8—S1	0.4 (3)	C4—C5—C6—C7	−178.5 (2)
C9—N2—C8—N1	−178.83 (18)	C4—C5—C6—C1	−0.9 (3)
C8—N2—C9—C10	151.8 (2)	C1—C6—C7—O1	−140.9 (2)
C8—N2—C9—C11	−86.2 (3)	C5—C6—C7—O1	36.7 (3)
C6—C1—C2—C3	0.4 (3)	C5—C6—C7—N1	−143.55 (19)
C2—C1—C6—C5	0.1 (3)	C1—C6—C7—N1	38.9 (3)

### Hydrogen-bond geometry (Å, º)

**Table:**

*D*—H···*A*	*D*—H	H···*A*	*D*···*A*	*D*—H···*A*
N1—H1*N*···S1^i^	0.81 (2)	2.66 (2)	3.4439 (19)	165 (2)
N2—H2*N*···O1	0.87 (2)	2.00 (3)	2.662 (2)	132 (2)

Symmetry code: (i) −*x*+1, −*y*+2, −*z*.
