# Supplementary figures and images for: Crystal structure of N-(propan-2-yl­carbamo­thio­yl)benzamide
Source: Acta Crystallogr E Crystallogr Commun. 2015 Jan 1;71(Pt 1):o56–7. doi: 10.1107/S2056989014027133 (PMC4331893; doi:10.1107/S2056989014027133)

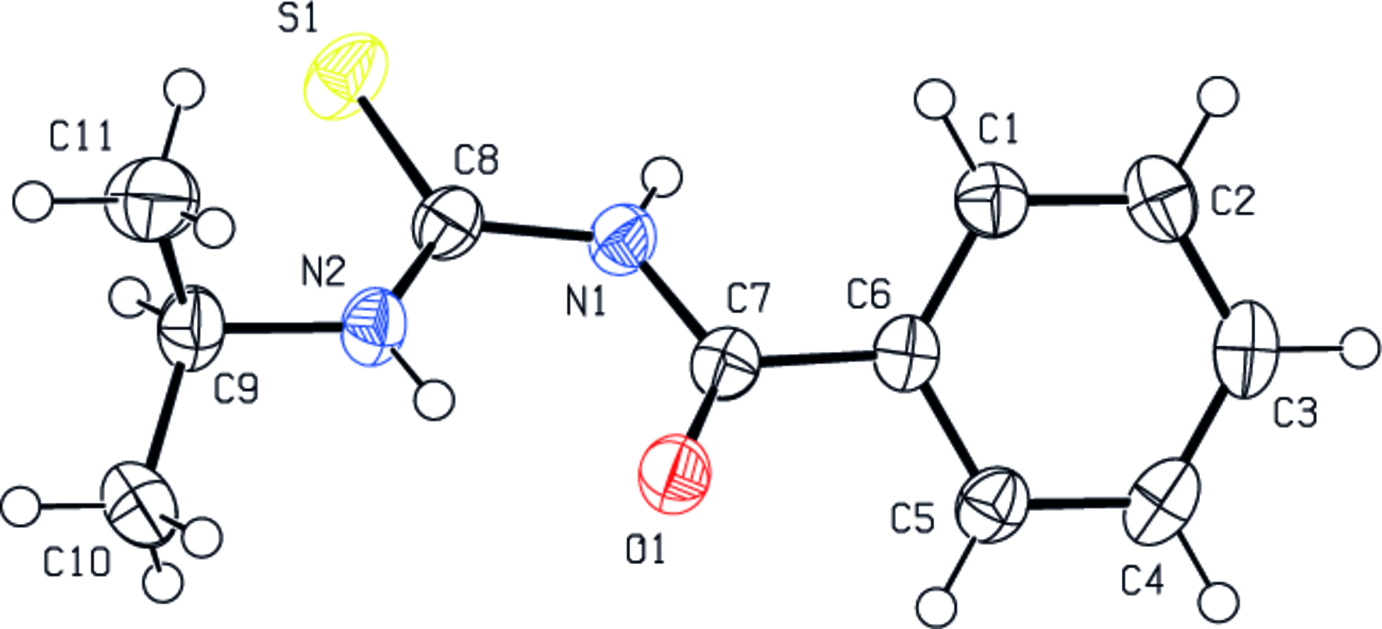

Supplement: Supplementary file 4 [file e-71-00o56-fig1.tif]

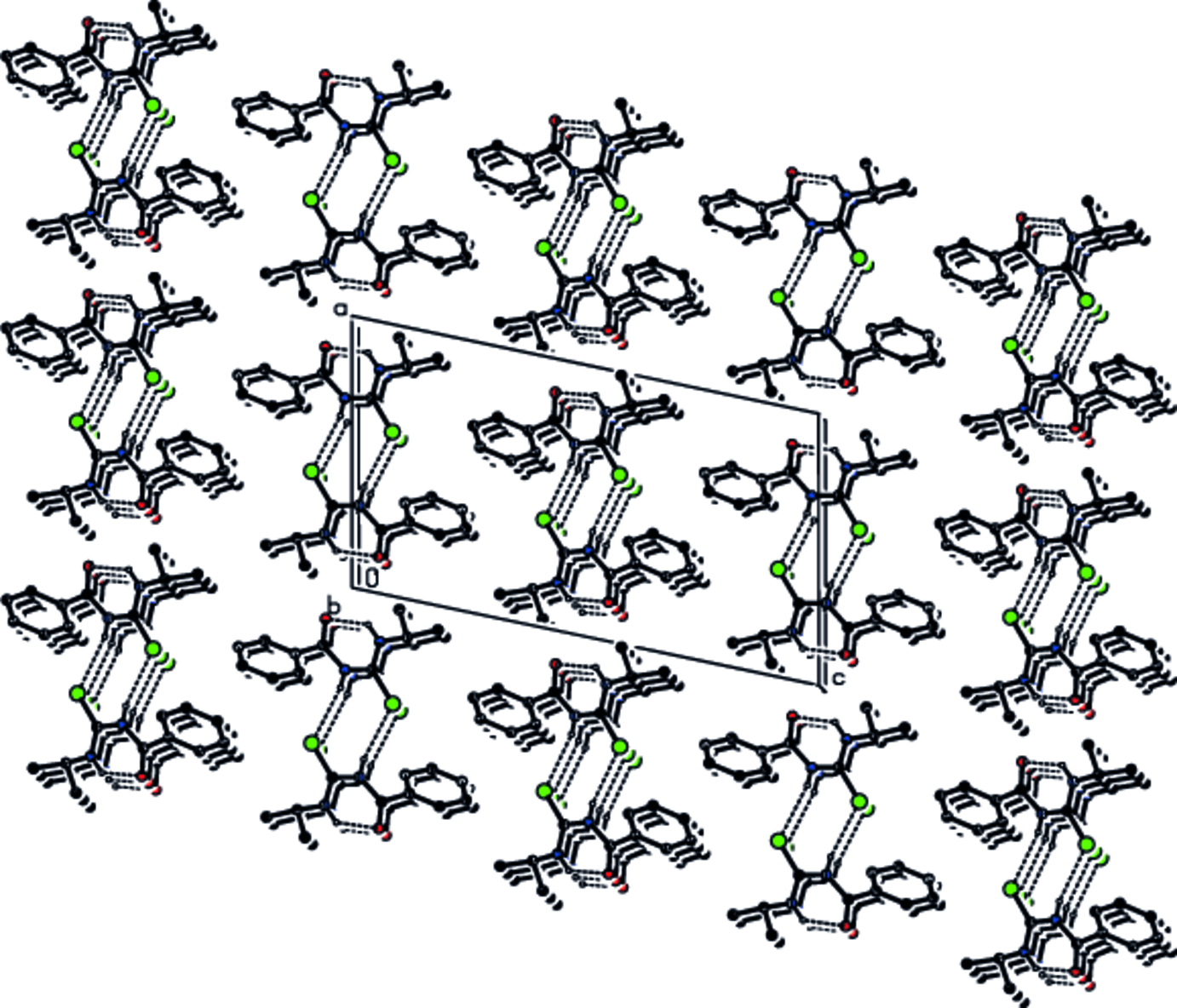

Supplement: Supplementary file 5 [file e-71-00o56-fig2.tif]
